# Mental health, smoking and poverty: benefits of supporting smokers to quit

**DOI:** 10.1192/bjb.2020.88

**Published:** 2020-10

**Authors:** Vicky Salt, Ciaran Osborne

**Affiliations:** 1 Action on Smoking and Health, UK

**Keywords:** Smoking, poverty, inequality, physical health, education and training

## Abstract

Smoking rates among people with common mental health conditions remain around 50% higher than those in the wider population; this is a significant cause of the 10–20-year reduced life expectancy of people with mental health conditions. However, the effects of smoking go far beyond physical health. Research estimates that smokers with mental health conditions could be spending as much as £2200 a year on tobacco, pushing an estimated 130 000 people with a common mental disorder into poverty. The Government has set a target for England to be smokefree by 2030; however, without a dramatic increase in support, smokers with mental health conditions risk being left behind. Action on Smoking and Health provides the secretariat for the Mental Health & Smoking Partnership. The Partnership aims to reduce the inequality in smoking rates between people with mental health conditions and the wider population. It brings together Royal Colleges, third-sector organisations, trade unions and academia to review progress and highlight areas for further action.

## The challenge

People with mental health conditions die on average 10–20 years earlier than individuals in the wider population.^1^ High smoking rates are one of the key reasons for this health inequality.^2^ Smoking rates remained largely static among people with mental health conditions during the 20 years from 1993 to 2013, at around 40%.^3^ Although new figures suggest that this prevalence is starting to decline, smoking rates among people with common mental health conditions remain around 50% higher than those in the wider population.^4,5^

However, the effects of smoking go far beyond reduced life expectancy; it influences employment prospects,^6^ reduces earnings,^6^ increases care needs^7^ and causes poverty.^8^ Higher smoking rates are associated with every indicator of disadvantage, but most adult smokers want to quit.^9^ Smokers with mental health conditions are aware of the risks to people around them from second-hand smoke and see this as a motivator to quit; clinicians must offer support to enable them to do so.

The challenge of The Mental Health and Smoking Partnership is to help tackle these inequalities.^10^ The Partnership was established in 2016 following publication of *The Stolen Years: The Mental Health and Smoking Action Report* by Action on Smoking and Health (ASH), with the ambition of reducing smoking prevalence to 5% or less among people with mental health conditions.^11^ The report sets out the sector-wide action needed to reduce smoking rates among people with mental health conditions, from reviewing the training of the mental health workforce to building the research base on peer support and expert-by-experience-led interventions.

Three years on, the Government has published the Tobacco Control Plan for England,^12^ NHS Long Term Plan^13^ and Prevention Green Paper,^14^ committing to reducing smoking prevalence among people with mental health conditions. However, change is not happening quickly enough. The recent Green Paper *Advancing our Health: Prevention in the 2020s* sets an ambition to end smoking in England by 2030, defined as a prevalence of 5% or less.^14^ To achieve this, using the latest publicly available data,^15^ it is estimated that the annual rate of decline from 2014–2030 among people with mental health conditions needs to be an ambitious 1.82 percentage points a year, more than double the previous rate of decline. Unless we dramatically increase the support available, this target will not be met, and people with mental health conditions will be left behind.

## Tobacco, health and poverty

In his landmark 2010 report, *Fair Society, Healthy Lives*, Michael Marmot made clear the moral dimension of tackling health inequalities: ‘Inequalities are a matter of life and death, of health and sickness, of well-being and misery. The fact that in England today people from different socioeconomic groups experience avoidable differences in health, well-being and length of life is, quite simply, unfair and unacceptable’.^16^

Half of all lifetime smokers will die from their addiction, that is, more than 96 000 people every year in the UK. For every smoker who dies, an estimated 30 others will be living with smoking-related diseases.^17^ This should be reason enough to act.

However, there are also short-term, immediate benefits to quitting smoking. Smoking decreases disposable income (defined as household income after tax and National Insurance contributions), which can be returned to the household when smokers quit. Smokers in social housing spend, on average, 14.6% of their disposable income on tobacco, compared with 6.9% among owner occupiers and 5.8% of those living in privately rented accommodation.^18^ This has substantial economic consequences, with more than 1 million people – including 263 000 children – living in poverty as a direct result of income lost to expenditure on tobacco. The proportion of social housing tenants living in poverty increases from around a quarter (28.3%) to two in five (42.8%) once the costs of smoking are considered.^8^

Research commissioned by ASH and Public Health England has shown the extent to which people with mental health conditions are economically disadvantaged due to smoking.^19^ Higher rates of smoking are found among people with a mental health condition living in poverty (Table 1). Looking at the number of cigarettes smoked and taking into account purchases of illicit and cheap tobacco, researchers estimate that smokers in poverty spend an average of around £1200 a year on smoking, at 2015 prices.^19^ However, adjusting for underreporting of the amount of tobacco smoked, smokers with a mental health condition below the poverty line could be spending over £2200 a year on tobacco.^19^

**Table 1:** Smoking prevalence according to mental health disorder and poverty^19^

Type of disorder reported	Smoking prevalence in whole population	Smoking prevalence among those in poverty
Common mental disorder	34%	46%
Currently taking psychoactive medication	34%	46%
Longstanding mental disorder	40%	52%

Using the conservative estimate of £1200, researchers estimate that around 130 000 people with a mental health condition are pushed into poverty by expenditure on tobacco. Table 2 shows a breakdown of smokers with common mental disorders, those taking psychoactive medications and those with longstanding mental disorders across the UK, who are pushed into poverty by expenditure on tobacco.^19^

**Table 2:** Population estimates of the number of UK adults with a mental disorder who are in poverty and who smoke, and who are drawn into poverty by their expenditure on smoking^19^

	Common mental disorder (CIS-R score >12)			Currently taking psychoactive medications			Longstanding mental disorder		
	Smokers with disorder who are in poverty^a^	Smokers with disorder who are in poverty^b^	Numbers with disorder drawn into poverty by smoking^a^	Smokers with disorder who are in poverty^a^	Smokers with disorder who are in poverty^b^	Numbers with disorder drawn into poverty by smoking^a^	Smokers with disorder who are in poverty^a^	Smokers with disorder who are in poverty^b^	Numbers with disorder drawn into poverty by smoking^a^
UK	1 217 733	904 647	135 304	553 801	405 282	55 380	717 110	425 420	100 062
England	1 021 983	759 225	113 554	464 778	340 133	46 478	601 835	357 034	83 977
Scotland	103 011	76 527	11 446	46 848	34 284	4685	60 662	35 987	8464
Wales	58 913	43 767	6546	26 793	19 607	2679	34 694	20 582	4841
Northern Ireland	33 825	25 128	3758	15 383	11 257	1538	19 919	11 817	2779

CIS-R, revised Clinical Interview Schedule; HBAI, households below average income.

a. Poverty based on 60% median gross income within survey.

b. Poverty based on HBAI poverty threshold.

The effects of expenditure on tobacco are compounded by lower earnings among smokers in employment, with non-smokers earning on average 6.8% more than smokers. Smokers are also significantly less likely to be in employment than never-smokers. Long-term smokers are 7.5% less likely to be in employment than never-smokers.

People with mental health conditions are more likely to be unemployed, to receive benefits and to be living in relative poverty than those without mental health conditions.^2,20^ It is well documented that debt and financial stress can exacerbate mental ill health and that people with mental health conditions are more likely to experience debt problems.^21^ The interactions between smoking, poverty, debt and mental health suggested by these studies are important reasons to support smokers to quit.

## Effects on mental health

Further motivation to support smokers with mental health conditions to quit comes from the positive influence this can have on mental health. Quitting smoking is associated with reduced depression, anxiety and stress, as well as improved positive mood and quality of life compared with continuing to smoke.^22^ For some people with mental health conditions, smoking can feel like ‘self-medicating’, and people say that it is an important way for them to deal with stress. However, this relief is temporary, linked to relieving withdrawal from nicotine and in the long term it can exacerbate symptoms.^23^ The effect of smoking cessation on anxiety and depression appears to be at least as large as that of antidepressants.^22^

Not only does smoking cessation improve physical health and reduce the chances of developing a smoking-related disease, supporting smokers to quit can also lift them out of poverty, return valuable disposable income to households and improve mental health.

It is also crucial that we remember that a majority of smokers want to quit.^9^ This is true among all smokers, including those with mental health conditions.^24^ An even larger majority (71%) of smokers wish they had never started.^25^

## Providing support to quit

This raises the question: are services doing enough to support smokers with mental health conditions to quit? In 2016, the Five Year Forward View for Mental Health set a target for all in-patient mental health services to be smokefree by 2018.^26^ This commitment was reiterated in the Tobacco Control Plan for England, which stated that it would include ‘providing integrated tobacco dependence treatment pathways’^12^ in line with National Institute for Health and Care Excellence (NICE) guidance: *Smoking: Acute, Maternity and Mental Health Services* (PH48).^27^ There are no specific targets for community mental health services, yet ASH's survey of community mental health practitioners, discussed below, shows that there is a need to focus on the support provided by community mental health teams.

### In-patient settings

This 2018 target has been missed. A 2019 survey of mental health trusts in England found that nearly one in five (18%) did not have a comprehensive smokefree policy in line with NICE guidance.^28^ Over half (55%) of trusts reported not always asking patients about their smoking status on admission, and 57% of trusts said that staff accompany patients on escorted leave to smoke every day, a practice which is: ‘*…outdated. It reduces the resources available to deliver clinical care*^29^ *and causes direct harm to patients*.’^12^

Further, although all trusts reported that they offer nicotine replacement therapy (NRT) to patients, only 49% offered varenicline (Champix).^28^ This is likely to be due in part to historic misunderstandings about the effects of varenicline on mental health. However, evidence shows that varenicline is not associated with negative outcomes for people with mental health conditions.^30–32^ As reflected in the Royal College of Psychiatrists’ position statement on prescribing of varenicline: ‘*varenicline is a generally safe and well-tolerated medication which has been proven to increase rates of smoking cessation in psychiatric and non-psychiatric populations*’.^33^

However, prescribing rates for varenicline are falling fast, particularly for people with mental health conditions. Primary care data for over 200 000 smokers shows that smokers with mental health conditions were 31% less likely to be prescribed varenicline than NRT, despite varenicline being more effective.^32^ Smokers with mental health conditions who were prescribed varenicline were 19% more likely to have successfully quit at 2-year follow-up, compared with those prescribed NRT.^32^

These policies must be addressed to ensure that all smokers are being offered the best support available to quit, in line with NICE guidance. Smokers with mental health conditions are often more heavily addicted,^2^ and ensuring they have access to the most effective pharmacotherapy is essential to supporting quit attempts.

ASH's survey also showed significant variation in approaches to vaping, from allowing a range of devices to be used across trusts, to prohibiting the use of e-cigarettes. National guidance from PHE,^34^ the Royal College of Physicians^35^ and the Royal College of Psychiatrists^33^ highlights that vaping is substantially less harmful than continuing to smoke. Under UK regulations, the Medicines and Health Care Products Regulatory Agency (MHRA) oversees notification of new nicotine-containing e-cigarettes and e-liquids, and operates a yellow card reporting system for any adverse reactions.^36^ A report is not proof that a reaction was caused by vaping, just that the reporter suspected it might have been. From 20 May 2016 through to 9 January 2020, the MHRA had received 84 yellow card reports on 245 adverse reactions, including 27 serious respiratory events.^37^ In 2019, over 3.6 million people in England were estimated to be using e-cigarettes.^38^

E-cigarettes are the most popular aid to quitting smoking in England,^39^ and research has shown that using an e-cigarette along with behavioural support can be twice as effective for quitting smoking compared with using NRT.^40^ Further, vaping is much cheaper than smoking. Research suggests that smokers who switch to e-cigarettes spend around £417 a year on vaping, substantially less than the estimated expenditure on tobacco of £1200 a year.^41^ These benefits should not be underestimated, and trusts should review policies in line with the latest evidence^37,42^ and national guidance.^43^

Smokers also want greater provision and variety of support. In a focus group with 12 participants with experience of in-patient services or with family in in-patient services, no participant felt that the support offered on admission to a smokefree in-patient service was sufficient.^44^ They noted that smokefree often felt like a ‘tickbox exercise’ rather than part of their care, with references to clear failings in the support options available: ‘*Handing someone a card with a number should not, in my opinion, count as actively giving someone smoking cessation support – that's what I've had. But they can say they've given you some support; the fact that you couldn't use the phone at the time doesn't seem relevant*’.

To reduce the inequality in smoking rates between people with mental health conditions and the wider population, it is essential to improve the support offered in in-patient services. There are examples of trusts leading the way on this agenda, offering excellent support and engaging with smokers to ensure that they are addressing the broader social aspects of smoking and not just physiological withdrawal. Public Health England's videos on implementation of NICE PH48 illustrate some of these examples.^45^ If we are to achieve a smokefree 2030 for people with mental health conditions, these examples must become the norm.

### Community services

A similarly patchy picture of support is seen in community services, which support the majority of people with mental health conditions.^46^ Although there is less evidence here, a small-scale, self-selecting survey conducted by ASH of 103 mental health nurses (representing 33 trusts) and 171 psychiatrists (representing 48 trusts) found that over 55% reported receiving no training on supporting smoking cessation.^47^ Only around a quarter of respondents said that they ‘always’ or ‘usually’ delivered very brief advice on smoking.

Prescribing medications for smoking cessation was reported to be similarly uncommon in community settings. Three-quarters (76%) of qualified nurses said that they never prescribed smoking cessation medications (NRT or varenicline), as did three-fifths (59%) of psychiatrists.^47^

## What works?

This absence of support is concerning, especially as there is a strong evidence base around what works to support smokers to quit. Smokers are three times more likely to quit successfully with the support of a specialist stop-smoking service than when attempting to quit unaided.^48,49^

The Smoking Cessation Intervention for People with Severe Mental Ill Health (SCIMITAR) pilot^50^ and randomised controlled trial (SCIMITAR+)^51^ were built on this evidence base. The SCIMITAR trials tailored the support set out in NICE guidance for people with mental health conditions, showing that such an approach is both effective and cost-effective. SCIMITAR compared the effectiveness of combined behavioural and pharmacological support for smoking cessation with usual care.^51^ Trial-condition participants received face-to-face behavioural support delivered by a trained mental health professional and prescriptions of their choice of smoking cessation medications, the most commonly chosen being NRT. Adaptations for people with severe mental health conditions – including extended pre-quit sessions, ‘cut down to quit’, and home visits – were offered in the trial arm. Compared with usual care, this intervention more than doubled quit rates at 6-month follow-up and showed significant improvements after 12 months.^51^

Participants in the SCIMITAR trial reported that the intervention being delivered by mental health nurses was important to them. It was important that they felt this support was being delivered by someone who would understand their mental health diagnosis without judgement and with professional expertise.

An ASH/Rethink focus group participant highlighted clearly the unique part a mental health nurse could play in supporting smokers: ‘*Community mental health nurses visit people in their own homes, and they see how that person is managing. Smoking's expensive … if it's the difference between paying your electricity bill and buying a packet of fags… If this person is struggling, the community mental health nurse is in a good position to advise and act as a sign-post … and link with GP services and the local chemists that offer smoking cessation*’.^44^

This illustrates the value of training mental health professionals to deliver smoking cessation advice and support. However, studies have shown that mental health staff may not see addressing smoking as part of their role. One study found that only 48% of respondents felt that addressing smoking was within their remit as a mental health professional,^52^ and one in five were not sure whether quitting smoking would have a positive effect on recovery or thought quitting smoking could have negative effects.^52^

The persistence of these myths undermines the potential for mental health services to support smokers to quit, and highlights the need for smoking and smoking cessation to be included in the training of mental health professionals.

## Making change happen

Population-level interventions that have driven down smoking rates nationally have largely failed to reach smokers with mental health conditions. Although the specific commitment to supporting people with mental health conditions to stop smoking in the NHS Long Term Plan is welcome, the pace of change is currently slow.^13^ Transformation funding through the NHS Long Term Plan will not be in place nationally until 2023–2024.^13^ Although this additional funding and national commitment is welcome, it will not, on its own, help enough smokers with mental health conditions to quit.

Smoking rates among people with mental health conditions will not reach the national ambition of 5% or less by 2030 without a trained workforce that sees smoking and smoking cessation as part of their role. The current lack of training in smoking cessation among the mental health workforce, as evidenced by trust and community surveys, is a problem that must be tackled if staff are to be able to deliver on these commitments.

Smokers expect doctors to ask them about smoking and deliver advice. If clinicians are not asking about smoking, it sends the signal that this is nothing to worry about. All psychiatrists should be able to deliver very brief advice, an evidence-based brief intervention on smoking that asks about smoking status, advises that support is available that will increase the chances of successfully stopping, and signposts smokers to further support. This is not designed to support someone through their quit attempt; it is about raising the issue and motivating smokers to try to quit.

Smokers with mental health conditions are likely to be more heavily addicted and therefore can find quitting harder.^2^ Ensuring that smokers with mental health conditions have access to the right pharmacotherapy to support them to quit is essential. Mental health trusts must ensure that the most effective treatments, including combination NRT and varenicline, are on their formularies and that they are being prescribed to patients in line with NICE guidance.^27,53^

Psychiatrists have a key role in reducing smoking among people with mental health conditions and in turn need appropriate training to deliver this. Although people can become immune to statistics about disease and death caused by smoking, reminding them about the effects that smoking has on well-being and quality of life is always worthwhile.

The number of people with mental health conditions pushed into poverty by smoking demands urgent action. Although quitting smoking will not solve poverty for everyone with a mental health condition, giving people the support they need to quit is a big step in the right direction.

## About the authors

**Vicky Salt** is Policy Manager at Action on Smoking and Health, Hatton Garden, UK. **Ciaran Osborne** is Interim Director of Policy, Action on Smoking and Health, Hatton Garden, UK.

## Supplementary material

For supplementary material accompanying this paper visit https://doi.org/10.1192/bjb.2020.88. click here to view supplementary material
